# Supplementary material for: Are carbon emissions trading and green financial instruments synergistic? -Comprehensive quantitative research based on content analysis
Source: PLoS One. 2024 Mar 7;19(3):e0298601. doi: 10.1371/journal.pone.0298601 (PMC10919601; doi:10.1371/journal.pone.0298601)
Supplement: S1 Data — (ZIP) [file pone.0298601.s001.zip › Data/地级市Z_F工作报告.zip!/╔╜╬≈/┬└┴║╩╨/└┤╫╘╣1⁄2╓┌║┼í╛┬φ┐╦╩2╛▌═°í┐_2007.docx]

各位代表：

现在，我代表市人民政府向大会作工作报告，请予审议，并请各位政协委员和其他列席人员提出意见。

一、2006年工作回顾

2006年是“十一五”规划的开局之年，也是我市提出并实施“三个发展”、“三大工程”和“双百双千”项目大会战的起步之年。一年来，在中共吕梁市委的正确领导和市人大、市政协的有力监督与支持下，市政府坚持以科学发展观为统领，紧紧抓住“两区”开发的重大机遇，按照“开放引进、开发拉长、多元做大、科学发展”的经济结构调整思路，开拓进取，扎实工作，全市经济社会在“十五”基础上，乘势而上，蓬勃发展，取得了新的成就。

——经济平稳较快增长，总体实力继续攀升。全市地区生产总值完成382.4亿元，增长18.9%；规模以上工业增加值完成182.5亿元，增长37.3％；财政总收入完成77.12亿元，一般预算收入完成25.67亿元，分别增长28.6%、37.66%，剔除探矿权、采矿权收入，有8个县在全省的位次前移，全市位次前移到第五位；社会消费品零售总额达到101亿元，增长20.71%；居民消费价格总水平为2.3%。地区生产总值、规模以上工业增加值、社会消费品零售总额等指标增幅居全省第一，市域经济总体实力进一步攀升。

——重点领域投资增势强劲，财政支出体现发展取向。全社会固定资产投资达到202.9亿元，增长32.01%。第一产业投资2.84亿元，增长3.2倍；水利、环境保护及公共设施管理业投资6.64亿元，增长39.73％；交通运输业投资27.49亿元，增长42.98%。市本级财政医疗卫生支出增长82.62％，最低生活保障支出增长15.52％，自然灾害生活救助支出增长35.37％，全市用于“三农”方面的支出达到27.74亿元，增长35.71%，统筹发展、和谐发展迈出了新步伐。

——基础设施建设成效显著，社会事业发展步伐加快。全市城镇化率达到33.23％，建成区绿化覆盖率达到29.7％，城市集中供热普及率达到45％；每百平方公里公路密度达到62.9公里；人口自然增长率控制为6.65‰；高中阶段毛入学率增加到54.9％，高等教育毛入学率达到24％。这些指标的增幅均高于“十一五”规划的分年度要求。

——节能降耗力度继续加大，生态环境建设进展顺利。万元地区生产总值二氧化硫和化学需氧量分别减排21.14%和25.4％；矿区煤炭资源回采率达到55%，万元地区生产总值综合能耗下降5.8％，万元工业增加值综合能耗下降6.33%，万元地区生产总值平均耗水量下降12.5%。

——就业社保工作不断加强，城乡群众生活继续改善。新增城镇就业岗位2.76万个、下岗失业人员再就业1.84万人，城镇登记失业率为2.5%；各项社会保险综合参保人数达到77.8万人次，覆盖率达到65%；4个新型农村合作医疗试点县（市）参合农民达到128.2万人，参合率达到87.89％。城镇居民人均可支配收入9230.5元，增长13.5％；农民人均纯收入2462元，增长23.7％。

在这里，我向大会郑重报告，市一届人大四次会议通过的经济社会发展考核指标体系的46项指标均顺利完成！市政府向全市人民承诺的“十件实事”全部兑现！全市“十一五”规划的主要工作首战告捷！

一年来，我们主要抓了以下几方面的工作：

（一）扎实开展农业农村工作，全力推进扶贫开发和社会主义新农村建设

稳步推进农业结构调整。雁门关生态畜牧经济区所属项目建设步伐加快，优质核桃连片发展和红枣烘干房建设两项重点工作取得显著成效。粮食获得较好收成，东西两山小杂粮产业区种植面积达到292万亩，占粮田总面积的58.4%。特色种养业正在向区域化、专业化、规模化方向发展，肉蛋奶产量、牛猪羊存栏显著增长。

着力拓宽农民增收渠道。继续扶持龙头企业做大做强，不断加大劳动力培训和转移力度。年销售收入超1000万元、5000万元和亿元以上的龙头企业分别达到26个、5个和3个。新增劳务输出9.98万人，农民人均劳务收入566元，同比增加108元，产业增收的道路越走越宽。

全力推进扶贫开发工作。继续实施整村推进、扶贫移民等扶贫增收工程，完成55个村的整村推进和128个村的扶贫移民工作，有效缓解了边远特困山村的生存和生活困难。全市又有11.97万贫困人口的温饱问题得到解决，超额完成了年度任务。

全面启动社会主义新农村建设。出台并实施了资源型企业帮扶建设新农村和捆绑涉农项目、整合支农资金等政策性措施，扎实推进“十村示范、百村试点、千村治理”，深入实施农村生态家园富民工程，全面启动“万村千乡市场工程”建设，均取得了显著成效。

（二）全力实施“双百双千”项目大会战，不断掀起招商引资高潮

充分放大“两区”开发的政策效应，全面铺开“双百双千”项目大会战。本着严格管理、严格考核、严格奖惩的原则，出台并落实了一系列政策和制度。272个“双百双千”项目已立项186个，开工170个；“项目推进年”活动全面提速，年初确定的100个重点工业项目，累计完成投资125亿元，已建成投产39个，工业经济的规模和水平实现了新的提升。

进一步提高对外开放水平，招商引资成为经济工作一大亮点。在“港洽会”、“沪洽会”、“中博会”、“厦洽会”、“乌洽会”等招商引资活动中共签订合同、协议59项，引资45亿美元、158.3亿元人民币，又有20多户市外知名企业落户吕梁，对外开放的裂变效应正在逐步显现。积极创新项目融资方式，在太原成功举办“吕梁政银企对接会”，与14家省级金融机构签订贷款协议、意向1303亿元，占到“双百双千”项目总投资的一半以上。

（三）继续加快产业结构升级步伐，着力转变经济增长方式加速推进传统产业规模化进程

有效利用市场拉动、行政推动两方面的力量，积极实施大项目牵动战略，一批煤炭、钢铁、电力、化工、建材等行业的大项目相继启动，有力促进了工业经济规模化发展。全市规模以上工业企业比上年净增63户；产值上亿元的工业企业达到87户，净增19户；32户列入市“三个梯队”的重点企业工业总产值占全市的63.9％。

继续强化主导产业园区式集聚。交城夏家营、文水百金堡和孝义梧桐工业园区被国家发改委、国土资源部核准为省级经济开发区。全省对外经济合作重点项目—晋沪吕梁煤化工业园区各项工作进展顺利。建成和在建的工业园区（项目区和示范区）达到12个，入园企业新增24户，达到183户，列入统计范围的七大工业园区完成工业增加值69.7亿元，占全市工业增加值的38.2%；实现利税17.2亿元，占全市工业企业利税总额的28.8％。

着力推动资源循环利用。立足资源比较优势和产业发展实际，坚持循环经济发展理念，推动产业循环式组合，引导企业循环式生产，着力改造提升煤炭、焦化、冶金三大传统支柱产业，加快培育发展煤化工、电力、铝镁、新型材料等新兴支柱产业，一批以针状焦、苯酐、炭黑、甲醇、烯烃等为重点的煤化工项目以及汽车电器、特种玻璃项目先后开工建成。循环经济正在逐步走上加快发展的轨道。

（四）紧紧扭住基础设施建设不放松，大力加快特色城镇化建设

交通方面，209国道、苛大线、祁方线等干线公路改造基本完成，307国道改扩建工程部分完工；离军高速公路进展顺利，今年年底正式通车；沿黄干线公路和沿黄旅游公路建设全面启动；新增通水泥（油）路的行政村144个，建制村通客车率达到86.9%；吕梁机场前期工作进展迅速，已被列入国家民航总局“十一五”机场建设规划。太中(银)铁路吕梁山隧道工程进展顺利，吕临支线前期工作有序推进；孝柳铁路正在进行电气化改造，太古岚铁路已完成可研，阳侯地方铁路正式奠基,岢瓦铁路今年可望建成投运。我市交通运输状况、区位条件即将发生重大改善。

水利方面，横泉水库枢纽工程提前竣工，柏叶口水库已被列入国家“十一五”水利发展计划，千年水库、魏家沟水库、坪底水库、阳湾子水库以及枣区提黄灌溉等骨干水源工程正在大力推进；引文入川工程管沟掘进和管道安装正紧张施工，年内可投入使用。

电力方面，柳电二期建设进展顺利，三期工程已取得所有支持性文件，被列入山西省“十一五”电力建设规划；500千伏输变电站可研报告通过初审；以兴县2×60万千瓦、石楼2×60万千瓦、离石2×30万千瓦为主的22个重大电源项目顺利推进，一批电网项目相继开工或建成，电力紧张的局面即将得到较大缓解。

城市建设方面，利用国家开发银行这一融资平台，大力度投入，相继在市区铺开15大项35小项重点市政工程，城市功能和城市品位继续提升。以创建卫生文明城市为目标，全面引深“城市管理年”活动，建成了20座免费公厕，增配了清运设备，充实了环卫队伍，城市环境卫生得到明显改善，已被命名为省级卫生城市。孝义、汾阳两市和10个县城的城市经营和管理水平有了新的提高，部分重点城镇发展特色鲜明，辐射带动能力进一步增强。

（五）始终坚持统筹发展理念，努力实现社会事业发展新突破

“普九”成果稳步提高，“双百”示范学校创建工程深入实施，农村中小学现代远程教育工程全面启动，“两免一补”政策落实到位，105个城镇中小学校标准操场建设任务全面完成。普通高中办学水平明显提高，高考达线人数同比净增617人，增幅居全省第一。撤并农村小学375所，建成农村寄宿制学校54所、明德小学16所。职业教育和成人教育稳步推进。教育发展环境进一步优化。吕梁学院（吕梁煤炭职业技术学院）和凤山教育园区建设进展顺利。

科技投入力度继续加大，高新技术得到大力推广，专利申报量增幅明显，每10万人专利申请数达到3.74项。以信息化带动工业化，全市新型工业化产值达到120亿元，新型工业化水平达到30%。

文化建设全面加强。电视连续剧《吕梁英雄传》等一批文学艺术精品获奖，市青年晋剧团赴京演出获得成功。非物质文化遗产申报和文物保护工作成效显著。文化市场监管力度加大，知识产权保护得到加强。文化下乡活动蓬勃开展，专业文化院团整体实力步入全省前列，皮影、剪纸、木偶等传统文化产品走向国内外大市场。

乡镇卫生院改造工程全面展开，县、乡、村三级卫生服务网络日趋完善，公共卫生体系建设成效明显。新型农村合作医疗覆盖面进一步扩大，284万农业人口已有近一半开始受惠。

全民健身运动广泛开展，竞技体育水平提高，一批运动健儿在各类比赛中成绩优异。吕梁体育馆经营管理水平提高，相继举办了一批高级别赛事，吕梁游泳馆开工建设。

（六）高度注重解决民生问题，稳步推进社会主义和谐社会建设

新一轮的就业再就业政策全面落实；企业离退休人员基本养老金按时发放，失业、医疗、工伤等保险待遇全部兑现，共发放各项社会保险金4.6亿元；劳动争议仲裁案件全部得到解决，为农民工追讨拖欠工资1688万元。城乡低保、社会救济、优抚安置等工作得到扎实推进。

环境保护力度继续加大，准入门槛进一步提高，污染性项目“三同时”制度得到较好遵从，清洁生产大范围实施，单位生产总值主要污染物排放量明显下降；“一企一矿绿化一山一沟”治理模式广泛推广，造林绿化“六大工程”进展顺利，“蓝天碧水”工程取得新的成效。

以煤矿安全生产为重点的大安全监管力度继续加大，制度建设体现出重大成效。信访稳定“百日大会战”成果丰硕，市、县、乡三级信访稳定长效机制基本建立，群体访、重复访明显下降，发展环境有了新的改善。

（七）继续加强社会主义民主法制建设，全力提升依法行政水平

主动接受人大监督，积极支持政协参政议政。自觉坚持向人大及其常委会报告工作，认真听取并积极办理人大代表、政协委员的提案、议案，答复各类意见、建议、批评等548件，答复率为100%。

高度重视政府法制工作，深入贯彻《行政许可法》。进一步清理并公布了行政许可项目和非行政许可(审批)项目，市政府新的政务大厅投入运行，一站式、便民化、高效率的服务得到广泛认同。“平安吕梁”建设继续引深，“打黑除恶”专项行动和“百日冲刺大会战”打出了气势，打掉了犯罪分子的嚣张气焰。

加强机关行政效能建设，大力开展政风行风评议活动。效能监察工作全面加强，市、县两级职能部门公开作出服务承诺，对部分不作为或乱作为的单位和个人作出了严肃处理。政风行风评议活动深入开展，参评单位多向拓展，政务环境明显改善，对外形象有了新的提升。

以上成绩的取得，是省委、省政府正确领导和大力支持的结果，是全市人民在中共吕梁市委的领导下，同心同德、拼搏进取的结果。在此，我代表市人民政府，向辛勤工作在全市各条战线上的广大工人、农民、知识分子、干部，向驻吕全体解放军指战员、武警官兵和公安干警，向各民主党派、无党派人士和各人民团体，向所有关心、支持吕梁经济社会发展的朋友们，表示衷心的感谢和崇高的敬意！

各位代表，“十一五”的良好开局，展现出我市经济社会发展的美好前景，但发展过程中存在的主要问题和薄弱环节也必须正视：一是经济增长方式总体依然粗放，能耗和环境压力仍然很大；二是农民增收的产业基础还不稳固，新农村建设进展很不平衡；三是社会事业欠帐很大，统筹发展的任务十分艰巨；四是防范安全生产事故的长效机制还不牢固，安全生产压力依然不小；五是投资和建设环境需要进一步优化，自主创新能力亟待加强；六是一些地方的社会治安不好，急需整治。以上问题，需要我们在今后的工作中认真加以解决，今年务求实现较大突破，务求取得显著成效，决不辜负人民的期望！

各位代表，生动的实践告诉我们，在吕梁这样一个百业正举的新兴城市，要实现好中求快、又好又快发展，必须坚持以科学发展观为统领，全力推进经济社会、人口资源环境、城乡之间、区域之间以及不同产业之间的协调发展；必须立足市情，着眼于加快发展、整体崛起，以强烈的责任意识和忧患意识，奋发图强，提高政府的公信力、执行力，充分调动各方面的力量，上下同心投入到工作实践中；必须坚持项目牵动，围绕提升产业层次、增强企业竞争力和改善发展环境等战略重点和薄弱环节，布局和建设一批重大项目，通过重点突破带动整体推进；必须坚持大开放、大引进，依托资源优势，引进和吸收一切有利于加快发展的要素，激活后发优势；必须坚持安全第一、预防为主、综合治理的方针，构建以煤矿安全为重点的安全生产长效机制，保障市域经济健康运行，维护国家和人民生命财产安全；必须始终着眼于以人为本、关注民生这一根本目标，切实把事关人民群众切身利益的问题解决好。我们坚信，只要全市上下励精图治，锐意进取，更加勤奋扎实地工作，就一定能够战胜各种艰难险阻，吕梁经济社会各项事业一定能够沿着科学发展的轨道不断前进！

二、2007年经济社会发展的目标任务

2007年是我市全面落实科学发展观，实现“又好又快”发展，加快构建和谐吕梁的重要之年。市政府工作的总体要求是：坚持以科学发展观为统领，不断引深“三大工程”建设，全力推进“双百双千”项目大会战，继续坚持“开放引进、开发拉长、多元做大、科学发展”的经济结构调整思路，抓住“两区”开发机遇，加快新型工业化、特色城镇化步伐，深化改革，扩大开放，创优环境，关注民生，统筹城乡，努力实现经济社会又好又快发展，积极推进和谐吕梁建设，以优异的成绩迎接党的十七大的胜利召开。

按照上述总体要求，今年我市经济社会发展的主要预期目标是：地区生产总值突破500亿元，全社会固定资产投资突破300亿元，工业增加值达到270亿元，社会消费品零售总额达到110亿元，外贸进出口总额达到5亿美元，财政总收入确保95亿元、力争突破100亿元大关，其中一般预算收入达到30亿元，城镇居民人均可支配收入达到10500元，农民人均纯收入达到2760元，居民消费价格总水平涨幅控制在3％以内，万元生产总值综合能耗下降6％，万元生产总值二氧化硫、化学需氧量各下降10％，城镇新增就业岗位25万个，城镇登记失业率控制在3％以内，市区环境空气质量二级以上天数达到215天。

根据上述总体要求和预期目标，今年将重点抓好以下十个方面的工作：

（一）以“双百双千”项目为载体，全面推进经济结构优化升级

着眼于产业链条延伸，做大做强传统支柱产业。原煤行业，着力改造一批有发展潜力的煤矿，巩固一批生产稳定的煤矿，淘汰一批不达标的小煤矿，不断加快“矿井大型化、企业集团化、产业链条化”进程。重点支持300万吨以上特别是1000万吨以上大型矿井建设，严格按照省政府“六条标准”对非法违法生产矿井实施关闭，力争年底使矿区煤炭资源回采率达到58%以上。焦化行业，严格落实“焦炭产量负增长”要求，以孝义梧桐、交城夏家营、汾阳三泉、离柳煤焦区四大焦化基地为重点，加大整合力度，优化生产布局，双向延伸产业链条，稳步提高行业的整体竞争力。冶金行业，以提高效益、减少污染为目标，严格限制普钢产能，关停取缔落后炼铁高炉，鼓励和支持发展高档次普钢和特种钢，依托大企业、大集团，开发适销对路的下游产品，打造煤-焦-铁-钢-材产业链。

着眼于产业多元发展，加快培育新兴产业。煤化工业，进一步完善煤化工技术路径和产业模式，培育一批在全省乃至全国具有影响的骨干企业和煤基化工研发中心，鼓励支持醇醚燃料、烯烃、苯胺等下游深加工产品发展，大力延伸产业链条。积极开发和利用煤层气、高炉尾气和焦炉煤气。加快晋沪吕梁煤化工园区建设步伐，促进煤基化工规模化、集聚化、集约化发展。铝镁工业，依托铝土矿资源优势，在富水区发展铝及铝材加工业。加大技术引进力度，对镁生产工艺进行技术改造，开发高性能、高质量、高附加值的镁合金深加工产品，逐步延伸煤电铝（镁）产业链。积极配合中铝、鲁能集团做好兴县、岚县氧化铝及深加工项目的前期工作，争取年内开工。新型材料工业，注重规模扩张，大力调整产品结构，依法淘汰落后产能，积极发展高标号水泥、特种水泥和轻质高温耐火材料，限制实心粘土砖生产，鼓励新型墙体材料生产，重点支持优质浮法玻璃、煤矸石制砖、粉煤灰建材制品等综合利用项目，促进新型建材产业做大做强。服务业，运用现代经营方式和信息技术，对商贸、餐饮、住宿、房地产等传统服务业进行改造，逐步形成层次分明、布局合理、功能齐全、连接城乡的市场集群，创造更多的就业岗位，有效增加从业者收入。积极引进大型物流企业，构建以太军高速公路和307国道为中轴的多功能、多层次的现代物流网络。大力发展通信和互联网服务业，推进吕梁电子商务网络建设。鼓励发展工程咨询、资产评估、法律、会计、审计、质量认证等知识密集型中介机构。旅游业，进一步挖掘优势旅游资源，引导社会资金参与景区开发，加快重点旅游县、镇、村基础设施建设，重点打造“三线四大特色精品”，逐步形成旅游产业集群，带动餐饮、商贸、建筑、文化等相关产业发展。

着眼于产业升级换代，大力发展高新技术产业。以煤炭、焦炭、钢铁、建材、铝镁等支柱产业为切入点，广泛引入和利用新型适用技术，发展资源节约型、产业科技型经济。加强自主创新能力建设，加快引进和开发能够促进传统产业优化升级的共性技术和关键技术，重点培育一批综合性较强、专业化水平较高的创业服务中心，带动主导产业由外延扩张型向内涵增长型转变。

着眼于产业集聚发展，加快工业园区建设步伐。坚持分类指导、区别对待的原则，严格准入条件，创新管理模式，完善功能设施，推进园区规范化建设、市场化服务、企业化管理进程，发挥园区集聚企业、循环利用、节约资源、集中治污的功能，促进主导产业集中、集群、集约发展。

（二）以发展现代农业为着力点，继续加大扶贫攻坚力度，加快社会主义新农村建设进程

加速推进农业产业化进程。继续完善以龙头企业为依托，农村经济合作组织为纽带，龙头带基地，基地联农户，产加销一体化的产业化发展模式，加速推进以“一区三片”为重点的特色农业功能区和农业科技园区建设，大力发展优质小杂粮、草食畜、干鲜果、蔬菜等特色产业，支持“绿色产品”和知名品牌拓展市场，促进传统农业向现代农业转变。高度重视气象事业对减轻气候灾害和促进农业增效、农民增收的功能和作用，积极发展避灾农业。加快60个“双百双千”农业项目建设步伐，年内新增产值超亿元的农业产业化龙头企业1个，新增超5000万元的2个，新增超1000万元的5个。

加速推进扶贫攻坚步伐。按照“进一步解决温饱、巩固成果、缩小差距、促进和谐”的扶贫开发新定位，继续实施整村推进、扶贫移民、劳动力转移培训和产业化扶贫四大“扶贫增收”工程，特别是要紧紧扭住转移培训、打造品牌、跟踪服务三个重要环节，提高劳务输出组织化程度，实施好农村劳动力转移“双五十万”工程和“阳光工程”，大力推进劳务经济产业化发展。

加速推进社会主义新农村建设。按照“5485”的总体发展目标，大力实施“三大覆盖工程”和“十村示范、百村试点、双百推进、千村治理”工程，突出规划编制、产业培育、环境整治、政策落实，确保农民增收基础不断夯实，农村环境明显改善。继续抓好资源型企业帮扶新农村建设、捆绑涉农项目、整合支农资金等重要政策措施的落实，确保新农村建设投入有较大幅度的增加。加快“生态家园富民工程”推进步伐，支持农村养殖场建设大中型沼气设施，支持农户兴建小型沼气工程。全面启动以农业生产资料连锁配送、农产品市场购销、农村消费品现代经营、再生资源回收利用为重点的“新网工程”，新建试点村便民店117个，铺开推进村便民店226个。认真抓好农民素质提升工程和科技入户工程，积极推进乡镇文化站建设，提高农民素质，培养新型农民，提升农村文明水平。

（三）以路水电建设为突破口，进一步缓解基础设施瓶颈制约

超常规推进交通建设。发挥好6亿元开行贷款的带动效应，继续加大县乡公路建设和养护力度，推进“村村通”工程向边远山村延伸。离军高速公路、沿黄干线公路吕梁段、石清二级公路及沿黄扶贫旅游公路兴县至柳林段等在建公路年内全线建成通车。开工建设汾孝大道和离石至中阳、离石至信义一级公路，完成离临柳石扶贫路离石至碛口段、307国道离石至冀村段、209国道横泉水库改线段、岢大线三交至大武段、桃临线交口段等改扩建工程。力争开工建设太(原)佳(县)、汾(阳)平(遥)两条高速公路。全面开工太中(银)铁路吕梁段建设工程，吕临支线立项开工；加快阳侯、太古岚、孝柳等地方铁路建设和改造步伐，完成苛瓦铁路建设任务。吕梁机场确保年内开工。

产业化推进水利建设。以“6+1”骨干水利工程建设为龙头，发挥政府主导作用，用市场化思路谋划重点水利工程建设。引入战略投资者，重点推进横泉水库城市、工业供水和农业灌溉工程，力争早日受益；柏叶口水库要加快前期工作步伐，争取早日立项开工；千年水库已经正式开工；坪底水库今年6月份之前开工；加大引文入川工程的协调和推进力度，确保年内竣工。抓好中小型水库除险加固工程。继续实施水土保持工程，年内新建淤地坝300座以上。

大力度推进电力建设。积极争取国家和省的支持，加快大型坑口电厂、热电联产机组及煤矸石、中煤、煤层气综合利用发电项目建设步伐，尽快实现输煤变输电的能源转型。积极推动柳电三期以及兴县、石楼2×60万千瓦和岚县、离石、交城2×30万千瓦等22个电源项目尽快上马。开辟电网建设“绿色通道”，大力支持省电力公司和山西国际电力公司投资的吕梁电网建设,加快500千伏输变电工程及6座220千伏输变电工程等电网建设与改造步伐，力争年内实现每县都有一座220千伏变电站，保障电网安全运行。

（四）以中心城市拓展提升为龙头，着力加快特色城镇化进程

优化规划，着力提升城市建设水平。突出自然文化特色，进一步完善市区总体规划，认真编制和完善控制性详规、修建性详规和各类专业规划。要十分珍惜和科学利用规划区建设用地，严管容积率，盘活国有地产，高标准抓好“城中村”改造，提高城市建设投入的经济和社会效益。

项目推动，着力完善城市功能。继续实施“三山”绿化和“一街两路(龙凤大街和滨河两路)”绿化提升工程，巩固提高城区绿化成果，年内建成区绿化水平提高到31.1％。加快城市污水处理厂和生活垃圾处理厂投运步伐，着力削减建成区环境污染。加快北川河综合整治、高速公路东西出口改造、滨河北路东延、东川河沿岸景观提升及城区段全程蓄水工程等重大市政工程的建设进度。完成城区集中供热二期工程，年底集中供热普及率达到52％。

强化管理，着力提升城市品位。加强城市公用设施建设与管理，大力发展市区公共交通，支持吕梁汽车站新站建设，规划建设一批短途客运停靠站（点）。大力度实施“市民素质提升行动”，加大市容市貌综合治理力度，巩固和提高城市卫生和文明水平。

多管齐下，着力增强城镇综合承载和辐射带动能力。继续提升孝义、汾阳两市和10个县城的建设与管理水平。注重内涵，体现特色，鼓励引入市场机制，加快重点城镇的绿化、污水处理、集中供热和供气等公用设施建设步伐，强化城镇交通拥挤、环境污染、食品安全、社会治安等突出问题的治理，提高城镇的档次与品位，带动周边农村加快发展。年内全市城镇化率达到34%。

（五）以深化改革扩大开放为动力，不断增强经济发展活力

深化各项改革，冲破加快发展的体制机制障碍。巩固和扩大税费改革成果，推进以乡镇机构改革为重点的农村综合改革，继续落实县乡财政体制改革、商贸粮食流通体制改革、民营水利改革和集体林权制度改革等改革措施，充分调动一切有利于农业农村全面发展的积极因素。理顺国企监管体制，落实“一退两置换”政策，加快以产权制度改革为核心的国企改革步伐。积极探索和完善对重点领域和薄弱环节的投融资体制改革，改善政府投资管理模式，完善政银企对接的长效机制，深化农村金融改革，着力解决财政投资效益低和中小企业融资难的问题。实施企业上市工程，争取有1—3户企业在境外上市，实现我市企业上市零的突破。

继续扩大开放，凝聚又好又快发展的更多力量。坚持全面开放、择优引进，以项目为载体，扩大领域，拓展深度，尤其要把着力点放在引进煤化工、现代采掘业、优质钢、大型坑口及综合利用发电、农副产品加工、高新技术等项目和技术上，重点在城市公用事业、基础设施建设、旅游景区开发及高级经营管理人才等方面的引进上求得突破。

大力发展民营经济和混合所有制经济。认真落实《中小企业促进法》，创优民营经济发展环境，加快中小企业投融资平台和担保体系建设，在投资核准、融资服务、土地使用、对外贸易和经济技术合作等方面，对各种所有制企业实行同等待遇，从资源、能源、运力、出口等指标配置方面给重点企业必要的倾斜，充分发挥民营经济促进市域经济跨越发展的重要作用。

（六）以环境保护和节能降耗为切入点，努力建设资源节约型和环境友好型社会

大力推进节能降耗工作。对新项目实行节能评价制度，认真落实节能降耗责任制，强化节能行政监察和执法监督，突出抓好重点行业和企业的节能减排工作，加大对违法违规企业的惩处力度，确保节能降耗指标全面实现。落实对节能降耗企业的税收优惠政策，加大对高耗能企业税费征管的力度，用有效的行政手段引导、鼓励企业节能减排。

继续引深环境保护攻坚战。目前，攻坚决战已经取得阶段性成果，175户严重违法排污企业已被关停，列入省政府分类处置名单中的企业正在实施严格整治，争取尽早解除“区域限批”，并以此为契机，全面推进以焦化企业为重点的环保专项整治，以实际行动和攻坚成果回报吕梁人民对我们的信任和支持！让全市人民早日呼吸上新鲜空气，喝上干净的水!

坚决淘汰落后生产能力。认真执行国家产业政策，完善市场准入、差别电价和税费征收等政策约束机制，加快淘汰落后工艺和产品，为优势企业腾出资源、腾出产能、腾出环境容量。引导和支持被淘汰企业转产、技改或新上符合国家产业、环保政策和我市产业发展规划的新项目。继续完善有利于产业素质提升的政策扶持措施，对具备煤气回收、焦油加工、废渣利用、节约资源条件的企业，在办理经营许可证、电力供给、铁路运力安排上给予倾斜支持。

严格执行耕地和资源保护政策。全面加强耕地管理，严格保护耕地特别是基本农田。千方百计盘活存量土地，最大限度地节约城乡建设用地，优先保障基础设施重点工程和产业发展重点项目以及社会公共事业项目的建设用地。加大土地整理、复垦与改造力度，确保全市耕地总量占补平衡。加强矿产资源保护与开发，加大对非法采矿的查处力度，节约利用好矿产资源。

大力推进造林绿化六大工程。继续加大造林绿化工作的力度，全面完成高速公路、国省道干线公路、铁路两侧的通道绿化任务，强力推进交通沿线荒山绿化、村镇绿化、厂矿区绿化、环城绿化和城市绿化，支持孝义市跨入全省园林城市行列。加大退耕还林、天然林保护、三北防护林等国家重点工程建设，努力开创“生态吕梁”、“宜居吕梁”建设新局面。全年植树造林382万亩。

（七）以经济社会持续协调发展为着眼点，全力推动社会事业快速发展

优先发展教育事业。全力推进义务教育标准化建设，完善农村义务教育经费保障机制，积极推进农村中小学现代远程教育工程，不断提高现代教育技术装备水平。坚持抓好农村“双百”示范校建设，进一步整合农村教育资源，新建农村寄宿制学校60所。继续推动示范高中建设，促进普通高中多样化发展，高中阶段毛入学率达到60%。坚持实施“科教兴县、兴乡、兴村”工程，狠抓农村实用技术培训和劳动力转移培训，积极探索工学结合、校企合作的实用人才培养模式，构建面向社会、面向市场办学的职业教育发展新格局。大力发展民办教育，重视并抓好学前三年教育普及工作。加快吕梁市区学校建设和改造步伐，着力缓解中小学班容量大的问题。加快吕梁学院建设步伐，年内完成建筑群主体工程。

大力发展科技事业。加大科技投入，倾斜扶持科技研发中心和技术创新基地建设，重点在煤焦化升级、新型能源和原材料开发、特色种植养殖和加工等方面取得较大进展。加快科技推广步伐，积极申报并力争有更多的项目列入国家和省级规划。加强自主研发，培育自主知识产权，进一步提高产业科技素质、人才科技素质以及科技对经济增长的贡献率。年内每10万人专利申请数达到4项，高新技术产业增加值占地区生产总值的比重达到3%。

加快发展卫生事业。加强公共卫生体系建设，完善传染病报告和预警机制，着力提高疾病预防控制能力；完善县、乡、村三级卫生服务网络，着力提高处理突发公共卫生事件的能力。进一步加大对农村卫生事业的投入，充分发挥乡镇卫生机构的枢纽作用。支持特色中医事业发展，推进农民健康教育和社区卫生工作。继续扩大新型农村合作医疗试点，新增6个试点县，让更多的农民群众享受到政策实惠。深化医疗卫生体制改革，推进医疗机构达标上等，规范医疗纠纷投诉处理行为，加强行业自律约束和医德医风建设，让广大群众就医方便而舒心。

继续推进文化、体育、广播电视等社会事业的改革发展。强化基层文化服务体系建设，挖掘传统文化内涵，繁荣地方特色文化，开发精品文化产业，通过文化艺术让全国乃至全世界了解吕梁、关注吕梁、投资吕梁。深入开展多层次、多形式的竞技运动和全民健身活动，提高体育竞技水平，增强人民群众体质。承办好第六届全省农民运动会。突出农村广播电视“村村通”、城市电视数字化和知识产权保护等工作重点，进一步加快广播电视、新闻出版、档案信息化以及文物保护、人口与计划生育、老龄等其他社会事业的发展。

（八）以构建和谐吕梁为目标，切实解决事关民生民安的实际问题

建立健全促进社会公平正义的制度体系。综合运用经济、法律、行政等多种手段，妥善协调社会各方面的利益关系，正确处理人民内部矛盾和其他社会矛盾，努力促进社会和谐。高度重视困难群众的生产生活问题，健全收入分配调节机制，努力缓解地区之间、城乡之间和社会成员之间收入分配差距扩大的趋势。继续加大对教育乱收费、药品价格虚高、公路“三乱”、农民负担反弹等现象的整治力度，严肃查处拖欠征地补偿费、拖欠农民工资、行政执法滥用强制手段、商业贿赂等违法违纪案件，妥善解决好关系群众切身利益的热点难点问题。整顿和规范市场经济秩序，严厉打击欺行霸市、商业欺诈等行为。

完善社会保障体系。进一步拓展就业渠道，抓好职业培训，提高就业技能，重点做好下岗失业人员再就业工作，积极帮助“零就业”家庭和就业困难人员就业，做好高校毕业生和复转军人就业工作。继续加大社会保险扩面工作，养老、失业、医疗、工伤等各项社会保险的综合覆盖率达到70％以上。认真落实城市和农村最低生活保障制度，继续实施社会救助制度，积极探索社会福利市场化运作机制，多渠道筹集资金，加快发展社会福利事业和残疾人事业，保障城乡群众特别是弱势群体的生活权益。整合教育救助资源，认真解决好贫困大学生上不起学的问题。

健全安全生产长效机制。树立“减量增安”理念，严厉打击非法违法企业，大力整合各类矿山企业，提高技术装备水平，落实安全生产制度，加大安全监管力度，有效防范道路交通、消防、食品安全、森林防火特别是重特大煤矿事故的发生。加快应急救援机构和队伍建设，完善应急预案，提高应对处置事故灾害的能力，最大限度地减少事故损失。

（九）以贯彻落实《依法行政实施纲要》为重点，切实加强民主法制和精神文明建设

积极推进社会主义民主建设。主动向人大及其常委会报告工作，自觉接受人大的法律监督和工作监督。密切同政协和各民主党派、工商联、无党派人士的联系。积极支持人民法院、检察院依法履行职责。充分发挥工会、共青团、妇联等群团组织的积极作用。继续做好民族、宗教、侨务、外事等工作。进一步完善基层群众自治组织和企事业单位民主管理制度，扩大政务公开、村务公开和厂务公开的范围和内容，保障人民群众的知情权、参与权、表达权和监督权。加强公务员队伍的建设与管理，落实事业单位改革措施，加大招才引智力度，为经济社会又好又快发展提供坚实的人才保障和智力支持。

全面落实依法治市措施。认真贯彻《依法行政实施纲要》，深入开展“五五”普法教育，努力增强公民的法制观念。搞好法律援助工作，维护妇女、儿童、残疾人、农民工等社会弱势群体的合法权益。全力推进“平安吕梁”建设，全面落实社会治安综合治理责任制，加强对重点地区和突出治安问题的整治，加大对基层政法机构基础设施建设投入，严厉打击各类刑事犯罪活动。完善信访稳定长效机制，强化矛盾排查调处工作，畅通群众诉求渠道，力争在“源头息访”上取得实质性成效。

切实加强社会主义精神文明建设。大力弘扬以爱国主义为核心的民族精神和以改革创新为核心的时代精神，在全社会树立共同的理想信念。深入开展以“八荣八耻”为主要内容的社会主义荣辱观教育，建立完善与社会主义市场经济相适应的社会公德、职业道德和家庭美德等思想道德体系。更加注重公务员队伍特别是领导干部的思想和作风建设，深入开展以倡导“八个方面”良好风气为重点的学习教育活动。大力推进文明和谐城市、文明和谐村镇、文明和谐单位等群众性精神文明创建活动，加强全民素质教育，培养公民高尚人格，促进和谐人际关系的形成。加强未成年人思想道德教育，引导青少年健康成长。继续加大科学知识普及力度，反对封建迷信，打击“黄赌毒”现象，倡导文明健康的生活方式。深入开展“双拥双服务”工作，推进人防工程建设，搞好国防教育和国防后备力量建设。

（十）以政府自身建设为保障，努力打造为民务实清廉高效政府

创新科学发展思路。树立科学的发展观和正确的政绩观，用科学行政、民主行政、依法行政的理念促进政府决策水平、管理水平和服务水平的提高。紧紧围绕“实现和谐发展、建设和谐吕梁”的宏伟目标，研究和完善总体工作思路，狠抓重点工作，加强薄弱环节，努力做到速度与结构、质量、效益相统一，经济发展和人口、资源、环境相协调。紧紧围绕“三大工程”建设和“双百双千”项目大会战的战略任务，以推进项目尽快开工、尽快投产达效为抓手，创新工作思路，强化制度约束，狠抓措施落实，全面夺取“双百双千”项目大会战的新胜利。

引深行政效能建设。坚持以改进机关作风、创优发展环境为目标，进一步转变政府职能，着力提高行政效能。始终把提高政府公信力、执行力作为创优发展环境的关键去抓。始终把深化行政审批制度改革作为创优发展环境的基础，加强市、县两级政务大厅建设，严格执行服务承诺制、首问负责制、限时办结制、超时默认制、责任追究制等制度，规范审批程序，净化审批环境，提高审批效率。始终把纠正行政不作为、乱作为和官僚主义、形式主义、弄虚作假现象作为改进作风、优化环境的着力点，切实加强对“双百双千”和招商引资项目落实情况以及环境保护、安全生产等重点领域的行政效能监察，确保政府机关高效运转，重点工作扎实推进。

完善考核制度体系。进一步强化责任意识、忧患意识和大局意识，自加压力，激发活力，严格考核，严格兑现，有令必行，令行禁止。着眼于全面落实省政府和市政府的庄严承诺、推进“双百双千”项目建设、完成各项考核指标等工作重点，建立和完善各项专门的考核奖惩制度，明确目标，细化责任，排出进度，定期考核，形成一级对一级负责、一级向一级交帐，一级抓一级、层层抓落实的落实机制。按照实现“又好又快”发展的要求，拓宽考核领域，全面考核经济、社会、自然、人文等综合指标的完成情况，推动各级领导干部转变传统的政绩观念。按照权责统一、依法有序、民主公开、客观公正的原则，加快建立以行政首长问责为重点的行政问责制度，并把行政问责与行政监察、审计监督结合起来，有责必问，有错必究，对损害人民群众利益的突出问题，严格依法追究责任。

倡导真抓实干作风。围绕今年全市的重点工作和未来发展的战略重点，深入开展专题调研和学习观摩，充分了解基层群众的呼声，学习借鉴先进地区的发展模式和宝贵经验，开动脑筋，创新思路，积极进取，加快发展。大兴求真务实之风，加大督促检查力度，凡是重大工作任务，认真做到事前有安排、事中有检查、事后有总结，坚决纠正以会议落实会议、以文件落实文件的官僚主义、形式主义歪风。

加强廉洁政府建设。牢固树立立党为公、执政为民的观念，始终牢记“两个务必”，常修为政之德，常思贪欲之害，常怀律己之心，把全心全意为人民服务的宗旨贯彻到各项工作和各个环节中。认真贯彻国务院、省政府和中纪委、省纪委廉政工作会议精神及廉洁自律的有关规定，不断加强世界观、人生观、价值观改造，牢固树立正确的权力观、地位观、利益观，坚持公平正义，不徇私情，正确行使党和人民赋予的权力，自觉筑牢拒腐防变防线。全面落实《建立健全教育、制度、监督并重的惩治和预防腐败体系实施纲要》和政府系统党风廉政建设责任制，严格执行会审、联签、民评和职务消费公示、公款报销公开、公务接待限额等“六项制度”，强化监察、审计等监督机关职能，从制度和源头上预防和治理腐败，坚决纠正部门和行业不正之风，坚决反对讲排场、摆阔气的奢侈浪费风气，以廉洁政府的形象取信于民。

各位代表，办好直接关系人民群众利益的好事、实事，是各级政府关注民生、顺应民意、凝聚民心的重要途径。市政府决定今年继续集中财力、精力，办好十件实事。一是努力缓解城乡居民看病难、看病贵的问题，完成36所乡镇卫生院改扩建工程，新型农村合作医疗制度试点县扩大到10个，试点县农民参合率达到90％，县级以上医疗机构药品价格平均下降30％左右。二是改善边远贫困地区学校办学条件，帮助42所农村寄宿制小学配置暖气。三是继续关心城乡特困群众生活，全面推行城市医疗救助制度，对94570名农村低保对象每年人均补助360元，对18370名符合条件的“五保”对象实行按标施保。四是积极推进工伤保险扩面工作，所有煤矿职工(包括农民工)全部参加工伤保险，非煤矿山、冶金、化工、建筑等高风险企业职工(包括农民工)90%以上参加工伤保险，参保人数达到10万人。五是继续改善农村生产生活条件，发展农村沼气用户2.13万户，解决25万农村人口饮水安全问题，新建通村水泥路（油路）1000公里，实现具备条件的建制村全部通公路。六是改善城镇困难居民住房状况，建设安康居住工程48万平方米，开工建设廉租住房2500平方米。七是帮助农民通过劳务输出、移民搬迁脱贫致富，完成引导性培训15万人，转移农村富余劳动力6万人，移民搬迁10500人。八是继续实施“万村千乡市场工程”，着力改善农村流通条件，新建3个配送中心、200个农家店，使农家店总数达到500个，覆盖全市70%以上的乡镇和20%以上的行政村；建设和管理并重，积极建设蔬菜种植基地，打击欺行霸市，切实解决“农民卖菜难、市民买菜贵”的问题。九是全面启动“信贷支农工程”，投放支农贷款130亿元，重点支持130个县域农业产业项目，创建130个省级信用村，扶持35个农民专业合作社，建设35个农村信用市场，支持35万信用户。十是切实推进节能降耗工作，着力改善生态环境，市区空气质量二级以上天数达到215天以上，万元地区生产总值综合能耗下降6%，万元地区生产总值二氧化硫、化学需氧量各下降10％。

各位代表，我们已经实现了“十一五”发展的良好开局。新的形势催人奋进，新的目标鼓舞人心，让我们更加紧密地团结在以胡锦涛同志为总书记的党中央周围，在中共吕梁市委的坚强领导下，锐意进取，开拓创新，励精图治，扎实工作，为建设经济繁荣、人民富裕、社会和谐、环境友好的新吕梁而努力奋斗!
